# Supplementary material for: Exploring gender-intentional implementation of Digital Health Information for Immunization in Ethiopia
Source: PLOS Glob Public Health. 2026 Apr 22;6(4):e0005530. doi: 10.1371/journal.pgph.0005530 (PMC13102215; doi:10.1371/journal.pgph.0005530)
Supplement: S1 Table — (DOCX) [file pgph.0005530.s001.docx]

# S1 Table Gender Integration in immunisation and Digital Health Policies in Ethiopia

| No | Title of the document | Type of document | Year | Issuing authority | Info on experts who prepared the document | Does the document assess the gender gaps as the core problem in the strategy/document? If yes, what common problems are explored? | Does the document present any strategy to address the gender divide/problem? If yes, what strategies are proposed? |
| --- | --- | --- | --- | --- | --- | --- | --- |
|  | [Ethiopia National Expanded Programme on immunisation COMPREHENSIVE MULTI-YEAR PLAN (2021-2025)](http://www.dataverse.nipn.ephi.gov.et/bitstream/handle/123456789/2941/cMYP%20%282021-2025%29.pdf?sequence=1&isAllowed=y) | Strategy | 2021 | Ministry of Health ​ | Developed by around 70 experts which above 55 were male | Yes, the document recognises that women and children are disproportionately affected by health disparities and there is limited intervention to address gender related barriers in immunisation. It highlights how gender inequalities impact access to healthcare services, including immunisation | yes, the document outlines several strategies to Reduce gender related barriers around immunisation practices by applying gender sensitive demand generation activities at all levels by the end of 2025 |
|  | National Implementation Guideline for Expanded Programme on immunisation | Guideline | 2021 | Ministry of Health | Developed by 26 male and 9 females | Not indicated | While there is no direct mention of gender-specific strategies, the guide outlines general strategies to ensure equitable access to vaccines |
|  | [Routine immunisation Catch-up Vaccination Guide lines](https://publications.jsi.com/JSIInternet/Inc/Common/_download_pub.cfm?id=25790&lid=3) | Guideline/Manual | 2022 | Ministry of Health | 22 experts, 20 male and two female | The document does not ex Not indicated | While there is no direct mention of gender-specific strategies, the guide outlines general strategies to ensure equitable access to vaccines |
|  | [Health Sector Medium-Term Development and Investment Plan (HSDIP)](https://arm.moh.gov.et/wp-content/uploads/2023/10/WBHSP_-EFY-2016-final.pdf) | Strategy | 2023 | Ministry of Health | Not specified | Yes, the document assesses gender gaps as a core problem. Common problems explored include: Gender disparities in health: Differences in health outcomes and access to healthcare services between men and women. Gender-based violence (GBV): The impact of GBV on women's health and well-being. Socioeconomic disparities: How socioeconomic factors intersect with gender to affect health outcomes | Yes, the document outlines several strategies to address the gender divide, including: Promoting Gender Equality in Health Services: Ensuring equitable access to health services for all genders. Strengthening Reproductive, Maternal, Neonatal, Child, Adolescent, and Youth Health (RMNCAYH): Focusing on improving health outcomes for women and children. Addressing Gender-Based Violence (GBV): Implementing programs to prevent and respond to GBV. Enhancing Women's Participation in the Health Workforce: Encouraging more women to join and advance in the health sector. Community Engagement and Empowerment: Involving women in community health initiatives and decision-making processes |
|  | [National gender mainstreaming manual for health](https://www.cmpethiopia.org/content/download/7153/27008/file/National%20Gender%20Mainstreaming%20Manual%20English%20version,12%20April%202021.pdf) | Manual | 2021 | Ministry of Health | 7 experts of which 6 were females | Yes, the identifies gender inequality as a key determinant of health. Common problems explored include: Gender-based stereotypes in health programs​ ,differences in access to health services due to sociocultural norms, Impact of gender roles on health-seeking behaviour​ ,gender norms influencing workload distribution and decision-making power within households​ | Yes, the manual provides several strategies to address gender gaps, including: Gender-sensitive policies to ensure equal access to healthcare​ , incorporating gender analysis in all health sector programs​ ,ensuring equal participation of men and women in programme design, implementation, and evaluation​ ,developing gender-sensitive indicators for monitoring and evaluation​ ,institutionalizing gender-responsive budgeting to allocate resources equitably |
|  | [Strategy for mainstreaming gender within digital health and health information systems in Ethiopia (2023/24-2025/26](https://dha.jsi.com/wp-content/uploads/2023/11/STRATEGY-FOR-MAINSTREAMING-GENDER.pdf) | Strategy | 2023 | Ministry of Health | 20 experts of which 5 were females | Yes, the strategy identifies gender gaps as a major issue in digital health and health information systems (HIS). Common problems explored include: Gender disparities in access to digital health technologies. Low digital literacy among women. Underrepresentation of women in decision-making platforms and HIS leadership. Limited gender-sensitive data collection and analysis. Cultural norms and biases affecting women's participation in health data systems | Yes, the document proposes several strategies, including: Ensuring a gender-balanced and competent HIS and digital health workforce. Integrating gender perspectives in HIS governance and policies. Promoting gender-responsive data management and decision-making. Addressing digital literacy gaps among women and female health workers. Strengthening partnerships and collaboration for gender mainstreaming |
|  | [Digital Health Blueprint](https://extranet.who.int/countryplanningcycles/sites/default/files/public_file_rep/ETH_Ethiopia_Digital-Health-Blueprint_2021.pdf) | Strategy/blueprint | 2021 | Ministry of Health, | 30 senior technical experts , gender not specified | While the blueprint does not explicitly frame gender gaps as a central issue, it acknowledges the importance of equitable access to digital health technologies. Some key gender-related concerns include: Limited digital literacy, particularly among marginalised populations, including women. Access barriers to digital health services due to sociocultural factors. Need for gender-sensitive health data and digital health workforce development | Yes, the document includes strategies that indirectly encourage gender-sensitive digital health workforce development, ensuring inclusive and equitable access to digital health services. |
|  | [Health Sector Transformation Plan II](https://www.globalfinancingfacility.org/resource/ethiopia-health-sector-transformation-plan-202021-202425) | Strategy | 2020 | Ministry of Health |  | Yes, recognises gender disparities in health access and outcomes. Progress is seen in gender mainstreaming and women’s empowerment in the health sector. Activities implemented include promoting empowerment of females in the health workforce, developing manuals on gender and orienting the health workforce. | Yes, the document considers various gender-focused interventions, including equitable distribution of the health workforce with a gender balance, mainstreaming gender across health programs and leadership, and promoting merit-based yet gender-equitable assignment of health facility leaders. |
