# Supplementary material for: Exploring gender-intentional implementation of Digital Health Information for Immunization in Ethiopia
Source: PLOS Glob Public Health. 2026 Apr 22;6(4):e0005530. doi: 10.1371/journal.pgph.0005530 (PMC13102215; doi:10.1371/journal.pgph.0005530)
Supplement: S2 Table — (DOCX) [file pgph.0005530.s002.docx]

**S2 Table** Illustrative participant quotes supporting key themes

*This document includes a minimal set of illustrative, fully de-identified quotes. All excerpts were selected in accordance with the informed consent obtained from participants and are covered under the study’s approved ethics and institutional review processes.*

| **Themes** | **Sub-Themes** | **Illustrative anonymized quote** |
| --- | --- | --- |
| Gender Dynamics in Digital Health Information for Immunization | Gender Understanding and Gender-Based Role Assignment in the Workplace | *Task assignment in the workplace is not commonly based on gender, and it is not an official practice or principle to allocate tasks according to gender. However, there are instances in the immunization sector where certain roles are specifically assigned to one gender, such as females being predominantly assigned to immunization service delivery at the community level. In contrast, within the community, there exists a norm and cultural practice of gender-based role assignment, often placing a disproportionate burden on females.* |
|  |  | *I haven’t had any previous experience or exposure to information specifically related to gender, nor am I fully aware of what it entails. In our health facility, there isn’t any specific consideration or policy that allocates tasks based on gender. Tasks are generally assigned without focusing on whether someone is male or female.* |
|  |  | *In the immunization program, as to my experience it is not common to focus on gender as a main consideration when planning activities. This is because we lack awareness and do not approach issues from this perspective. Tasks are simply assigned based on the available experts, without considering gender as a factor* |
|  | Gender Balance of Stakeholders Involved in Digital Health Information for Immunization | *As you know most vaccinators are Health extension workers in Ethiopia in which are females. Most of EPI focal in health centers are also females; you rarely find males in EPI at health center level. On the contrary woreda health bureaus are dominated by males. This is not done intentionally, but rather happens because most females do not usually qualify for the jobs in health bureaus. I am sure we will see a lot of women once they are able to meet the qualifications.* |
|  |  | *Most of the trust issues with men front line health service providers are deliberately set to be women. Regarding women empowerment, our office currently is developing leadership induction manual and conducting trainings which advocates for women to come to front as leaders. The trainings advocates and targets 50% or more women to be engaged in managerial and leadership roles at hospitals and health centers.* |
| **Gender Inclusiveness in Digital Health Information for Immunization** | Policy-makers’ perspective | *In the immunization program, both females and males participate at different levels, although females are the majority. Women are mainly concentrated at the immunization service provision level, with Health Extension Workers (HEWs) being exclusively female. This is because their primary target audience is mothers, who spend most of their time at home, where the majority of Health Extension Program (HEP) packages, including immunization, are implemented. However, at higher levels of the immunization hierarchy, such as supervision, leadership, and policymaking, female representation is much more limited. These roles are predominantly held by men, and policies, manuals, and guidelines are mostly developed by men, with minimal involvement from women.* |
|  | Digital health professionals’ perspectives | *There is no specific approach or intention to distribute digital health systems based on gender. Instead, the distribution is carried out based on individuals working in the area of service delivery. Similarly, capacity-building training activities are provided to eligible beneficiaries without considering the gender of the participants. We have no experience or practice in designing training programs specifically tailored to the gender of the recipients.* |
|  | Supervisors’ perspectives | *In my experience, I have had the opportunity to supervise vaccinators, and I observed that the majority-over 95%-are females. Both male and female vaccinators demonstrated good communication and interaction during supervision. However, there are some notable differences between them in terms of adhering to work principles and guidelines. Female vaccinators tend to excel in accepting feedback and translating it into actionable improvements compared to their male counterparts.* |
|  | Vaccinators’ perspectives | *I don't have different approaches to communicate with male and female caregivers. Most of the caregivers are mothers, so we mostly interact with them. Communication with fathers is rare, which leads to husbands having less understanding of child vaccination, its benefits, and possible side effects. Sometimes, when we call mothers, the husbands pick up the phone.* |
| **Gender-Intentional Data Management and Decision-making** | | *On the data management side, it’s a similar story. While there’s no significant difference in immunization coverage between male and female children, our DHIS2 administrative dataset doesn’t capture gender-disaggregated information. Gender-disaggregated data is only available through survey results. The DHIS2 platform aggregates data without breaking it down by gender, even though the source registers may include this information.* |
|  |  | *Access to and utilization of digital health tools in the immunization program are determined based on service delivery points and the staff assigned to those areas. There is no specific preference or criteria for distributing these tools among health workers based on gender. Similarly, capacity-building activities are conducted for the target users of the tools, without considering gender as a factor or parameter* |
| **Enablers of Gender-intentional inclusiveness Digital Health for Immunization** | | *In my view, a major facilitator for digital health is its status as a national priority. The government's strong commitment to digital transformation in the health sector guarantees equal access to these systems for everyone, regardless of gender. This prioritization fosters a conducive environment for implementing digital health tools, especially in immunization programs, while ensuring accountability and equitable resource allocation across all service delivery points.* |
|  |  | *Moreover, digital health has been established as a key priority within the health system, receiving dedicated attention and robust support from various collaborative partners.* |
| **Barriers to Gender-intentional Digital Health for Immunization** | | *In the immunization program, the concept of gender inclusiveness is not well understood, as you have described here. There are significant gaps in awareness and information among the actors across the health system. As a result, there are no specific interventions or activities in the immunization program implemented with a gender perspective. Instead, activities are cascaded in a uniform way by the available experts, without considering different approaches based on gender or designing interventions that target gender-related issues.* |
|  |  | *Additionally, there are challenges related to social norms, cultural factors, and even the perceptions of women themselves about taking on leadership and management roles in immunization programs. For instance, female representation in leadership and management is limited. One reason for this is that these roles demand extra effort, including tasks and meetings that often extend into the evening. This additional workload can be challenging for women, who typically have significant responsibilities at home. Many women feel that taking on leadership roles might affect their performance in both their professional and family lives. Let me share an example from my own experience. Both my husband and I are civil servants with demanding jobs. At one point, I was proposed for a director-level leadership position in the organization where I worked. However, after discussing it with my husband, we decided it was better for the family if I did not accept the position. This was because leadership roles require more time and commitment, and I wanted to ensure I had sufficient time for my family. In our society, while fathers play an important role, the role of mothers is often seen as irreplaceable due to biological and social factors* |
