# Supplementary material for: Exploring gender-intentional implementation of Digital Health Information for Immunization in Ethiopia
Source: PLOS Glob Public Health. 2026 Apr 22;6(4):e0005530. doi: 10.1371/journal.pgph.0005530 (PMC13102215; doi:10.1371/journal.pgph.0005530)
Supplement: Attachment — Submitted filename: Response_to_Reviewers20Jan2026.docx [file pgph.0005530.s004.docx]

20 January 2026

Dear Collins Otieno Asweto,

We are pleased to re-submit our revised manuscript “*Exploring Gender-Intentional Implementation of Digital Health Information for Immunization in Ethiopia*” (PGPH-D-25-03223) to PLOS Global Public Health after addressing the feedback that we received from reviewers and better alignment to journal requirements. We are grateful to the journal editors and reviewers for their time and constructive feedback.

The revised manuscript clarifies all of the request for more information from the reviewers. Details on each of the changes and any clarifications can be found in the point-by-point responses below.

Please do not hesitate to reach out if we can make additional changes to improve the manuscript. We look forward to hearing from you and thank you for considering this work for publication.

Sincerely,

Patricia Mechael

Journal Requirements:

1. Please provide a detailed online Financial Disclosure statement. This is published with the article. It must therefore be completed in full sentences and contain the exact wording you wish to be published.

a) State the initials, alongside each funding source, of each author to receive each grant. For example: “This work was supported by the National Institutes of Health (####### to AM; ###### to CJ) and the National Science Foundation (###### to AM).”

b) State what role the funders took in the study. If the funders had no role in your study, please state: “The funders had no role in study design, data collection and analysis, decision to publish, or preparation of the manuscript.”

For more information, please go to our submission guidelines:
<https://journals.plos.org/globalpublichealth/s/submission-guidelines#loc-financial-disclosure-statement>

**Financial Disclosure.** This work was supported by Gavi, the Vaccine Alliance (contract number HSIS 13318 8 23 to GA, BA, PM, XQ, MK, BE, KA, EL, SC, BT). The funders had no role in study design, data collection and analysis, decision to publish, or preparation of the manuscript.

2. Please ensure that the funders and grant numbers match between the Financial Disclosure field and the Funding Information tab in your submission form. Note that the funders must be provided in the same order in both places as well.

**Done**

3. Please update your online Competing Interests statement. If you have no competing interests to declare, please state: “The authors have declared that no competing interests exist.”

“The authors have declared that no competing interests exist.”

4. We note that your Data Availability Statement is currently as follows: “All data generated or analyzed during this study are included in this article and its supplementary information files”

Please confirm at this time whether or not your submission contains all raw data required to replicate the results of your study. Authors must share the “minimal data set” for their submission. PLOS defines the minimal data set to consist of the data required to replicate all study findings reported in the article, as well as related metadata and methods (<https://journals.plos.org/globalpublichealth/s/data-availability#loc-minimal-data-set-definition>).

For example, authors should submit the following data:

- The values behind the means, standard deviations and other measures reported;
- The values used to build graphs;
- The points extracted from images for analysis.

Authors do not need to submit their entire data set if only a portion of the data was used in the reported study.

If your submission does not contain these data, please either upload them as Supporting Information files or deposit them to a stable, public repository and provide us with the relevant URLs, DOIs, or accession numbers. For a list of recommended repositories, please see <https://journals.plos.org/globalpublichealth/s/recommended-repositories>.

If there are ethical or legal restrictions on sharing a de-identified data set, please explain them in detail (e.g., data contain potentially sensitive information, data are owned by a third-party organization, etc.) and who has imposed them (e.g., an ethics committee). Please also provide contact information for a data access committee, ethics committee, or other institutional body to which data requests may be sent. If data are owned by a third party, please indicate how others may request data access.

We are sharing a minimal dataset containing illustrative quotes from the qualitative data collection categorized by sub-theme.

5. If the reviewer comments include a recommendation to cite specific previously published works, please review and evaluate these publications to determine whether they are relevant and should be cited. There is no requirement to cite these works unless the editor has indicated otherwise.

No action needed

6. Please review your reference list to ensure that it is complete and correct. If you have cited papers that have been retracted, please include the rationale for doing so in the manuscript text, or remove these references and replace them with relevant current references. Any changes to the reference list should be mentioned in the rebuttal letter that accompanies your revised manuscript. If you need to cite a retracted article, indicate the article’s retracted status in the References list and also include a citation and full reference for the retraction notice.

No action needed

Additional Editor Comments (if provided):

Reviewers' comments:

Reviewer's Responses to Questions

**Comments to the Author**

1. Does this manuscript meet PLOS Global Public Health’s [publication criteria](https://nam02.safelinks.protection.outlook.com/?url=https%3A%2F%2Ftrack.editorialmanager.com%2FCL0%2Fhttps%3A%252F%252Fjournals.plos.org%252Fglobalpublichealth%252Fs%252Fcriteria-for-publication%2F1%2F010f019b8c113180-db962c22-948a-4d8d-8311-4ec49d87bee0-000000%2FN4EM5f_a8jIuC5KTDejrEIjZOW7uJCUMkrxsxhMPOmI%3D244&data=05%7C02%7Cpmechae1%40jh.edu%7Ca96b2979b25949db026a08de4c055896%7C9fa4f438b1e6473b803f86f8aedf0dec%7C0%7C0%7C639031783014439184%7CUnknown%7CTWFpbGZsb3d8eyJFbXB0eU1hcGkiOnRydWUsIlYiOiIwLjAuMDAwMCIsIlAiOiJXaW4zMiIsIkFOIjoiTWFpbCIsIldUIjoyfQ%3D%3D%7C0%7C%7C%7C&sdata=0W19M19hBwA4RfEW6KVAOojzqqgzMvuSSEdoRQn%2F0PQ%3D&reserved=0)? Is the manuscript technically sound, and do the data support the conclusions? The manuscript must describe methodologically and ethically rigorous research with conclusions that are appropriately drawn based on the data presented.

Reviewer #1: Yes

Reviewer #2: Yes

Reviewer #3: Yes

2. Has the statistical analysis been performed appropriately and rigorously?

Reviewer #1: N/A

Reviewer #2: Yes

Reviewer #3: N/A

3. Have the authors made all data underlying the findings in their manuscript fully available (please refer to the Data Availability Statement at the start of the manuscript PDF file)?

The [PLOS Data policy](https://nam02.safelinks.protection.outlook.com/?url=https%3A%2F%2Ftrack.editorialmanager.com%2FCL0%2Fhttps%3A%252F%252Fjournals.plos.org%252Fglobalpublichealth%252Fs%252Fdata-availability%2F1%2F010f019b8c113180-db962c22-948a-4d8d-8311-4ec49d87bee0-000000%2FTzLlFqGoQJLkhYZRJSdeyf9QN8XJGJDKxIv5ygxOLIg%3D244&data=05%7C02%7Cpmechae1%40jh.edu%7Ca96b2979b25949db026a08de4c055896%7C9fa4f438b1e6473b803f86f8aedf0dec%7C0%7C0%7C639031783014470033%7CUnknown%7CTWFpbGZsb3d8eyJFbXB0eU1hcGkiOnRydWUsIlYiOiIwLjAuMDAwMCIsIlAiOiJXaW4zMiIsIkFOIjoiTWFpbCIsIldUIjoyfQ%3D%3D%7C0%7C%7C%7C&sdata=%2F6KXovGX1%2FYfeYmM0RK9j45jbuDTwj1xg5zA45Imx0c%3D&reserved=0) requires authors to make all data underlying the findings described in their manuscript fully available without restriction, with rare exception. The data should be provided as part of the manuscript or its supporting information, or deposited to a public repository. For example, in addition to summary statistics, the data points behind means, medians and variance measures should be available. If there are restrictions on publicly sharing data—e.g. participant privacy or use of data from a third party—those must be specified.

Reviewer #1: Yes

Reviewer #2: Yes

Reviewer #3: Yes

4. Is the manuscript presented in an intelligible fashion and written in standard English?

PLOS Global Public Health does not copyedit accepted manuscripts, so the language in submitted articles must be clear, correct, and unambiguous. Any typographical or grammatical errors should be corrected at revision, so please note any specific errors here.

Reviewer #1: Yes

Reviewer #2: Yes

Reviewer #3: Yes

5. Review Comments to the Author

Please use the space provided to explain your answers to the questions above. You may also include additional comments for the author, including concerns about dual publication, research ethics, or publication ethics. (Please upload your review as an attachment if it exceeds 20,000 characters)

**Reviewer #1**: Manuscript Number: PGPH-D-25-03223
Exploring Gender-Intentional Implementation of Digital Health Information for Immunization in Ethiopia

General Comments:
The study titled “Exploring Gender-Intentional Implementation of Digital Health Information for Immunization in Ethiopia” is of great interest.
In the contemporary era of rapid technological advancement and shifting global demographics, women constitute a substantial proportion of the population worldwide and continue to serve as the primary caregivers and care providers. In the context of immunization, mothers traditionally bear the greatest responsibility for child healthcare decision-making and service utilization. However, the inclusion of fathers in immunization-related processes remains critically important to promote shared responsibility and improved health outcomes.
Therefore, the integration and implementation of a gender-intentional framework is an essential priority for immunization research and practice. The insights and lessons generated from this study will not only strengthen local immunization strategies but will also be applicable to other resource-constrained settings with similar sociocultural and health system contexts.
The paper is well-written scientifically.
I am recommending publishing this manuscript with minor changes.

Methods:
The process of reaching and recruiting participants could be further elaborated for future researchers.
Please clarify the methodological approaches used to minimize reporting bias and to account for the authors’ positionality and potential biases

Response: Thank you for this comment. Interview participants were purposely selected to satisfy the study’s qualitative objectives. Respondents were chosen based on their direct involvement in the development, implementation, supervision, or use of digital health information systems for immunization, ensuring that interviews focused on respondents with experience relevant to the research questions. To reduce reporting bias participants were selected across multiple levels of the health system, including national policy-makers, subnational managers, supervisors, and frontline service providers/vaccinators. This approach enabled triangulation of perspectives across roles with varying levels of responsibility. We have clarified the approach used for identifying respondents and interview participants in the Methods section.

**Reviewer #2**: Thank you very much for the opportunity to review this manuscript. This manuscript is well written and has an important topic for public health issues, especially immunization program. I have some comments to improve the quality of the manuscript.

Abstract
Authors stated DHIS2, please don’t use abbreviations in the first word

Response: This has been updated

Methods
Authors should describe in detail the key stakeholders involved in this study
Authors should add information how many participants in this study
Authors should explain detail each method conducted in this study such as a participatory design workshop, interviews, and stakeholders mapping. Who is participating in each method? Please explain them detail

Response: Thank you to the reviewer for flagging these sections that need more detail. Additional details have been included in the Methods section describing the participatory workshop, the stakeholder mapping and the selection process for key informant interviews. Individual characteristics of the stakeholders who participated in the interviews can be found in Table 1.

Results
Authors explained that they involved eight documents to conduct this study. Authors should mention them in detail

Response: The eight documents that were reviewed are detailed in Table S1. A new reference to this table is included in the Methods section to clarify where the reader can find these details.

In the subheading gender dynamics in digital health information for immunization, authors stated that Women’s involvement in digital health interventions development is minimal. Authors should add an evidence-based to support this statement.

Response: Thanks to the reviewer for this feedback. The statement was out of place and has been moved to the discussion section. The statement is supported by the findings from the stakeholder analysis which provides evidence for this statement (see section on Gender Balance of Stakeholders).

Authors stated that the gap is observed in DHIS2 system, which lacks a dedicated sex variable for the immunization program. As a result, immunization indicators cannot be analyzed and interpreted from a sex or gender perspective, even when source documents or registers capture sex disaggregated data. Could you clarify this statement? And please add the evidence to support this statement.

Response: We appreciate the reviewer’s feedback to clarify this paragraph. The section has been updated with more information about the flow of sex-disaggregated data that is not reported when data is entered into the national health information management system.

**Reviewer #3:** The overall study is highly significant considering that there is limited evidence around this. The author has made an excellent effort to address this issue.

1: The Introduction section can be elaborated more with detailed literature review & background.

Response: We appreciate the reviewer’s suggestion. The references and content of the Introduction and Discussion sections have been improved and updated to provide the reader with more details on the local context and gender-intentional approaches.

2: Line 46-54 can be readjusted and place it after line 55-61.

Response: While we appreciate the reviewer’s suggestion, relocating lines 46-54 would disrupt the narrative flow of the Introduction section; therefore, we have retained the original placement.

3: It would be good if the author mentions how many participants were approached and how many of them refused to take part in the study.

Response: Thank you to the reviewer for flagging this detail on respondent selection. Unfortunately we do not have documentation on the total number of key informants approached or the number who declined participation because key informants were selected using purposive sampling based on their predefined roles, expertise, and relevance to the study objectives. Recruitment was conducted through targeted invitations to individuals meeting these criteria, rather than through open or sequential sampling. As such, non-response or refusal was not systematically tracked. All individuals who participated provided informed consent before data collection. Clarification about the process of selecting key informants has been updated in the Methods section of the manuscript.

4: Inclusion of COREQ checklist is highly recommendable.

Response: We thank the reviewer for this suggestion. After consideration, we have not included the COREQ checklist as it is not required by the journal and the manuscript already reports all of the relevant qualitative methods and procedures in sufficient detail and in accordance with the journal’s guidelines.

6. PLOS authors have the option to publish the peer review history of their article ([what does this mean?](https://nam02.safelinks.protection.outlook.com/?url=https%3A%2F%2Ftrack.editorialmanager.com%2FCL0%2Fhttps%3A%252F%252Fjournals.plos.org%252Fglobalpublichealth%252Fs%252Feditorial-and-peer-review-process%2523loc-peer-review-history%2F1%2F010f019b8c113180-db962c22-948a-4d8d-8311-4ec49d87bee0-000000%2F2DI2Ipz48OECH8nSY1zN80a25SJcAHF6mt4uJroYCzI%3D244&data=05%7C02%7Cpmechae1%40jh.edu%7Ca96b2979b25949db026a08de4c055896%7C9fa4f438b1e6473b803f86f8aedf0dec%7C0%7C0%7C639031783014488600%7CUnknown%7CTWFpbGZsb3d8eyJFbXB0eU1hcGkiOnRydWUsIlYiOiIwLjAuMDAwMCIsIlAiOiJXaW4zMiIsIkFOIjoiTWFpbCIsIldUIjoyfQ%3D%3D%7C0%7C%7C%7C&sdata=vCeWpIpjac2NLN2rKhC%2FggruD77urlAP2cl848SZ4xM%3D&reserved=0)). If published, this will include your full peer review and any attached files.

**Do you want your identity to be public for this peer review?** If you choose “no”, your identity will remain anonymous but your review may still be made public.


For information about this choice, including consent withdrawal, please see our [Privacy Policy](https://nam02.safelinks.protection.outlook.com/?url=https%3A%2F%2Ftrack.editorialmanager.com%2FCL0%2Fhttps%3A%252F%252Fwww.plos.org%252Fprivacy-policy%2F1%2F010f019b8c113180-db962c22-948a-4d8d-8311-4ec49d87bee0-000000%2F7rjWx9iLwJ_CT23c7c4m35X8993tzmi8VRmWCpE99Lc%3D244&data=05%7C02%7Cpmechae1%40jh.edu%7Ca96b2979b25949db026a08de4c055896%7C9fa4f438b1e6473b803f86f8aedf0dec%7C0%7C0%7C639031783014509170%7CUnknown%7CTWFpbGZsb3d8eyJFbXB0eU1hcGkiOnRydWUsIlYiOiIwLjAuMDAwMCIsIlAiOiJXaW4zMiIsIkFOIjoiTWFpbCIsIldUIjoyfQ%3D%3D%7C0%7C%7C%7C&sdata=V5Lct0Xw%2F6sWN8OIvBMJEozNvWlR8STqpxmsgMQs3es%3D&reserved=0).

Reviewer #1: No

Reviewer #2: No

Reviewer #3: No

[NOTE: If reviewer comments were submitted as an attachment file, they will be attached to this email and accessible via the submission site. Please log into your account, locate the manuscript record, and check for the action link "View Attachments". If this link does not appear, there are no attachment files.]

Figure Resubmissions:

While revising your submission, we strongly recommend that you use PLOS’s NAAS tool (<https://ngplosjournals.pagemajik.ai/artanalysis>) to test your figure files. NAAS can convert your figure files to the TIFF file type and meet basic requirements (such as print size, resolution), or provide you with a report on issues that do not meet our requirements and that NAAS cannot fix.

After uploading your figures to PLOS’s NAAS tool - <https://ngplosjournals.pagemajik.ai/artanalysis>, NAAS will process the files provided and display the results in the "Uploaded Files" section of the page as the processing is complete. If the uploaded figures meet our requirements (or NAAS is able to fix the files to meet our requirements), the figure will be marked as "fixed" above. If NAAS is unable to fix the files, a red "failed" label will appear above. When NAAS has confirmed that the figure files meet our requirements, please download the file via the download option, and include these NAAS processed figure files when submitting your revised manuscript.
